# Crystal structure of 3-benzyl­sulfanyl-6-(5-methyl-1,2-oxazol-3-yl)-1,2,4-triazolo[3,4-*b*][1,3,4]thia­diazole

**DOI:** 10.1107/S2056989015017351

**Published:** 2015-10-03

**Authors:** Krishnaiah Vaarla, V. Rajeswar Rao, Mehmet Akkurt

**Affiliations:** a Department of Chemistry, National Institute of Technology, Warangal, Telangana 506004, India; b Department of Physics, Faculty of Sciences, Erciyes University, 38039 Kayseri, Turkey

**Keywords:** crystal structure, triazolo–thia­diazole system, isoxazole ring

## Abstract

In the title compound, C_14_H_11_N_5_OS_2_, the triazolo–thia­diazole system is essentially planar (r.m.s. deviation = 0.002 Å) and makes dihedral angles of 6.33 (12) and 42.95 (14)° with the planes of the oxazole and phenyl rings, respectively. In the crystal, face-to-face π–π inter­actions are observed between the thia­diazole and oxazole rings [centroid–centroid distance = 3.4707 (18) Å], leading to columns along [010].

## Related literature   

For the pharmocological properties of isoxazole, see: Kuz’min *et al.* (2007 ▸); Yermolina *et al.* (2011 ▸); Lilienkampf *et al.* (2010 ▸); Kamal *et al.* (2011 ▸). For the bioactivity of 1,2,4-triazoles coupled with the thia­diazole heterocylic ring system, see: Singh & Singh (2009 ▸). For biological applications, such as anti­microbial, anti­cancer, anti­viral and anti­helmentic properties, see: Habib *et al.* (1997 ▸); Bhat *et al.* (2004 ▸); Farghaly *et al.* (2006 ▸); Khalil *et al.* (1999 ▸). For the synthesis, see: Vaarla & Rao (2014 ▸). For a similar structure, see: Dinçer *et al.* (2005 ▸).
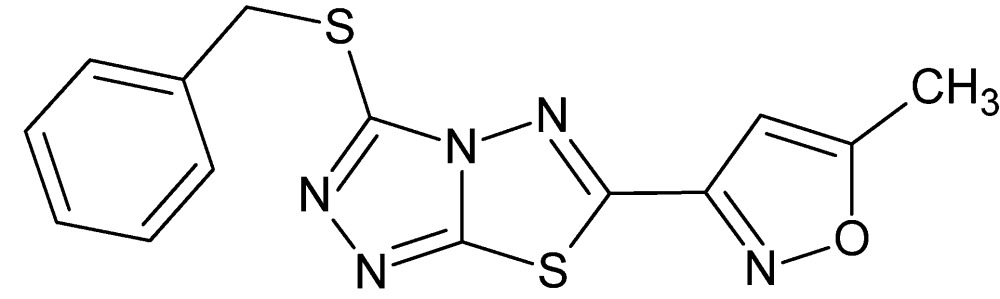


## Experimental   

### Crystal data  

C_14_H_11_N_5_OS_2_
*M* *_r_* = 329.40
Orthorhombic, 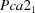

*a* = 16.271 (5) Å
*b* = 5.3804 (13) Å
*c* = 16.700 (4) Å
*V* = 1462.0 (7) Å^3^
*Z* = 4
Mo *K*α radiation
μ = 0.37 mm^−1^
*T* = 296 K
0.50 × 0.45 × 0.30 mm

### Data collection  

Bruker Kappa APEXII CCD diffractometer
Absorption correction: multi-scan (*SADABS*; Bruker, 1999 ▸) *T*_min_ = 0.836, *T*_max_ = 0.896
10433 measured reflections
3117 independent reflections
2905 reflections with *I* > 2σ(*I*)
*R*_int_ = 0.027

### Refinement  

*R*[*F* ^2^ > 2σ(*F* ^2^)] = 0.031
*wR*(*F* ^2^) = 0.077
*S* = 1.08
3117 reflections
200 parameters
1 restraint
H-atom parameters constrained
Δρ_max_ = 0.18 e Å^−3^
Δρ_min_ = −0.20 e Å^−3^
Absolute structure: Flack (1983 ▸)
Absolute structure parameter: 0.02 (2)

### 

Data collection: *APEX2* (Bruker, 2004 ▸); cell refinement: *APEX2* and *SAINT* (Bruker, 2004 ▸); data reduction: *SAINT* and *XPREP* (Bruker, 2004 ▸); program(s) used to solve structure: *SIR92* (Altomare *et al.*, 1993 ▸); program(s) used to refine structure: *SHELXL2014* (Sheldrick, 2015 ▸); molecular graphics: *ORTEP-3 for Windows* (Farrugia, 2012 ▸); software used to prepare material for publication: *WinGX* (Farrugia, 2012 ▸) and *PLATON* (Spek, 2009 ▸).

## Supplementary Material

Crystal structure: contains datablock(s) global, I. DOI: 10.1107/S2056989015017351/tk5386sup1.cif

Structure factors: contains datablock(s) I. DOI: 10.1107/S2056989015017351/tk5386Isup2.hkl

Supporting information file. DOI: 10.1107/S2056989015017351/tk5386Isup3.cml

**Fig. 1.**
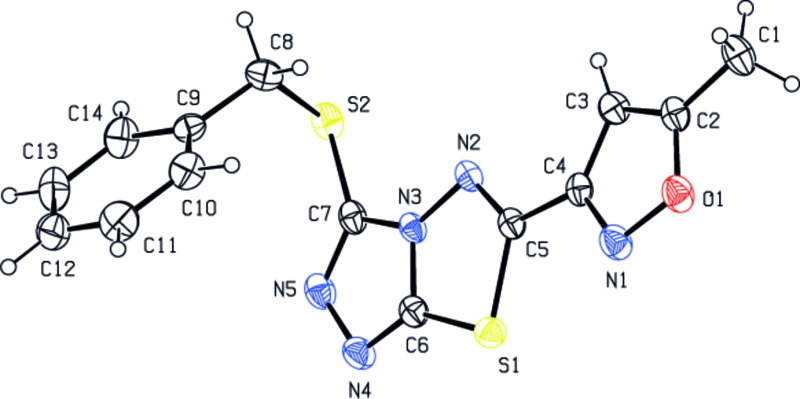
The title molecule with the atom numbering scheme. Displacement ellipsoids for non-H atoms are drawn at the 30% probability level.

**Fig. 2.**
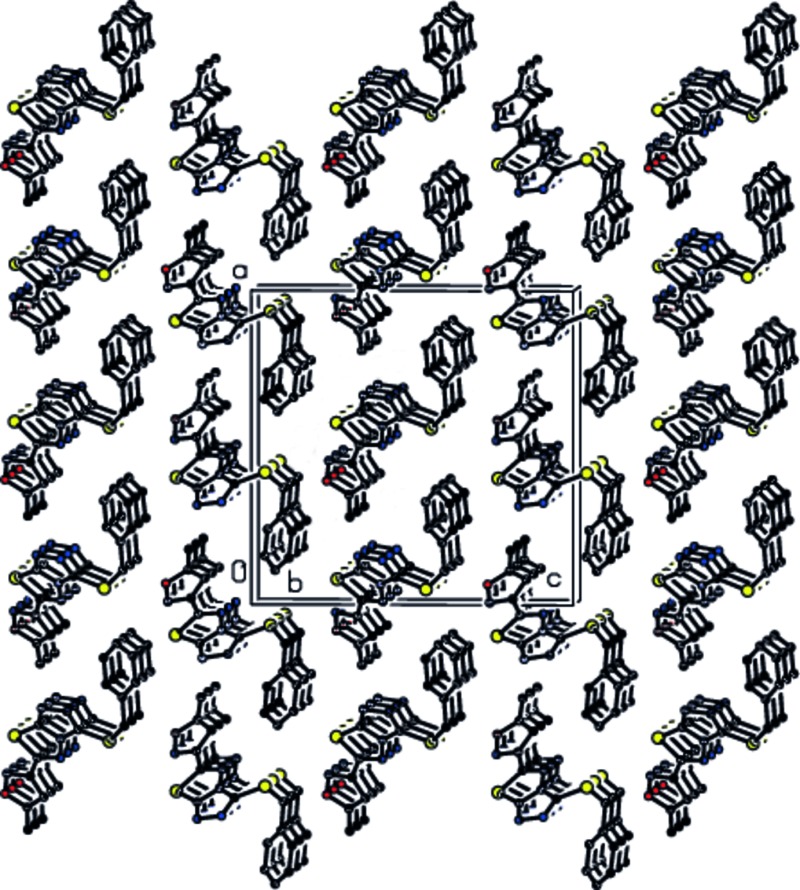
View of the molecular packing of the title compound down the b axis. All H atoms have been omitted for clarity.

. DOI: 10.1107/S2056989015017351/tk5386fig1.tif
The title mol­ecule with the atom numbering scheme. Displacement ellipsoids for non-H atoms are drawn at the 30% probability level.

b . DOI: 10.1107/S2056989015017351/tk5386fig2.tif
View of the mol­ecular packing of the title compound down the *b* axis. All H atoms have been omitted for clarity.

CCDC reference: 1425251

Additional supporting information:  crystallographic information; 3D view; checkCIF report

## supplementary crystallographic information

### S1. Comment

Nitrogen heterocyclic compounds have received much attention among researchers throughout the world for their applications as biological probes in the field of drug discovery. Isoxazole is a five membered O- and N-containing heterocylic compound and widely used as key building pharmacophore for drugs (Kuz'min *et al.*, 2007; Yermolina *et al.*, 2011; Lilienkampf *et al.*, 2010; Kamal *et al.*, 2011). Isoxazoles have a wide range of biological applications such as antiviral, anticancer, antibiotic, antituberculosis, antiinflammatory and antimicrobial agents, and as COX-2 inhibitors (Singh & Singh, 2009).

A large number of [1,2,4] triazolo[3,4-*b*][1,3,4]thiadiazoles have remarkable biological applications such as antimicrobial (Habib *et al.*, 1997), anticancer (Bhat *et al.*, 2004), antiviral (Farghaly *et al.*, 2006) and antihelmentic (Khalil *et al.*, 1999) properties.

The title molecule is shown in Fig. 1. The plane of the triazolo-thiadiazole system [r.m.s. deviation = 0.002 Å] forms dihedral angles of 6.33 (12) and 42.95 (14)° with the oxazole (O1/N1/C2–C4) and phenyl (C9–C14) rings, respectively. All the bond lengths and bond angles in the compound are within normal ranges and comparable with those reported in a similar compound (Dinçer *et al.*, 2005).

The crystal structure is stabilized by face-to-face π—π interactions [*Cg*1···*Cg*2 (*x*, -1 + *y*, *z*) = 3.4707 (18) Å and *Cg*2···*Cg*1 (*x*, 1 + *y*, *z*) = 3.4707 (18) Å] between the ring centroids, *Cg*1 and *Cg*2, of the thiadiazole and oxazole rings, respectively. Fig. 2 shows the molecular packing of the title compound down the *b* axis.

### S2. Experimental

The title compound was synthesized according to the published procedure (Vaarla & Rao, 2014). The compound was synthesized in two steps. In step one, a mixture containing equivalent amounts of 5-methylisoxazole-3-carboxylic acid and 4-amino-4*H*-[1,2,4]triazole-3,5-dithiol was refluxed in the presence of phosphorus oxychloride for about 5 h. After monitoring by TLC, the reaction mixture was cooled to room temperature and poured into crushed ice. The separated solid was filtered, dried and recrystallized from methanol.

In the second step the intermediate 6-(5-methylisoxazol-3-yl)-[1,2,4]triazolo[3,4-*b*][1,3,4]thiadiazole-3-thiol (1 eq.) was treated with benzyl bromide (1.1 eq.) in ethanol. The reaction mixture was refluxed for 5 h. After completion of the reaction, the reaction mixture was cooled to room temperature. The isolated solid product was filtered and washed with ethanol. Recrystallization was from ethanol.

### S3. Refinement

All H atoms were placed in calculated positions with C—H = 0.93 to 0.97 Å, refined in the riding model with *U*_iso_(H) parameters set to 1.2*U*_eq_(C) or 1.5*U*_eq_ (CH_3_ only). The (0 0 - 2), (2 0 1) and (2 0 0) reflections, whose intensities were affected by the beamstop, were removed from the final refinement.

### Crystal data

**Table:**

C_14_H_11_N_5_OS_2_	*F*(000) = 680
*M**_r_* = 329.40	*D*_x_ = 1.497 Mg m^−^^3^
Orthorhombic, *P**c**a*2_1_	Mo *K*α radiation, λ = 0.71073 Å
Hall symbol: P 2c -2ac	Cell parameters from 5610 reflections
*a* = 16.271 (5) Å	θ = 5.0–55.6°
*b* = 5.3804 (13) Å	µ = 0.37 mm^−^^1^
*c* = 16.700 (4) Å	*T* = 296 K
*V* = 1462.0 (7) Å^3^	Block, colourless
*Z* = 4	0.50 × 0.45 × 0.30 mm

### Data collection

**Table:**

Bruker Kappa APEXII CCD diffractometer	3117 independent reflections
Radiation source: fine-focus sealed tube	2905 reflections with *I* > 2σ(*I*)
Graphite monochromator	*R*_int_ = 0.027
ω and φ scan	θ_max_ = 28.4°, θ_min_ = 2.8°
Absorption correction: multi-scan (*SADABS*; Bruker, 1999)	*h* = −20→21
*T*_min_ = 0.836, *T*_max_ = 0.896	*k* = −6→7
10433 measured reflections	*l* = −16→21

### Refinement

**Table:**

Refinement on *F*^2^	Hydrogen site location: inferred from neighbouring sites
Least-squares matrix: full	H-atom parameters constrained
*R*[*F*^2^ > 2σ(*F*^2^)] = 0.031	*w* = 1/[σ^2^(*F*_o_^2^) + (0.0411*P*)^2^ + 0.1925*P*] where *P* = (*F*_o_^2^ + 2*F*_c_^2^)/3
*wR*(*F*^2^) = 0.077	(Δ/σ)_max_ = 0.001
*S* = 1.08	Δρ_max_ = 0.18 e Å^−^^3^
3117 reflections	Δρ_min_ = −0.20 e Å^−^^3^
200 parameters	Absolute structure: Flack (1983)
1 restraint	Absolute structure parameter: 0.02 (2)

### Special details

**Table:**

Geometry. Bond distances, angles *etc*. have been calculated using the rounded fractional coordinates. All su's are estimated from the variances of the (full) variance-covariance matrix. The cell e.s.d.'s are taken into account in the estimation of distances, angles and torsion angles
Refinement. Refinement on *F*^2^ for ALL reflections except those flagged by the user for potential systematic errors. Weighted *R*-factors *wR* and all goodnesses of fit *S* are based on *F*^2^, conventional *R*-factors *R* are based on *F*, with *F* set to zero for negative *F*^2^. The observed criterion of *F*^2^ > σ(*F*^2^) is used only for calculating -*R*-factor-obs *etc*. and is not relevant to the choice of reflections for refinement. *R*-factors based on *F*^2^ are statistically about twice as large as those based on *F*, and *R*-factors based on ALL data will be even larger.

### Fractional atomic coordinates and isotropic or equivalent isotropic displacement parameters (Å^2^)

**Table:**

	*x*	*y*	*z*	*U*_iso_*/*U*_eq_	
S1	0.58876 (4)	0.55792 (12)	0.27749 (5)	0.0430 (2)	
S2	0.56435 (4)	0.13539 (13)	0.56223 (5)	0.0480 (2)	
O1	0.41434 (13)	1.1668 (4)	0.26095 (13)	0.0513 (7)	
N1	0.47539 (15)	0.9833 (5)	0.26294 (16)	0.0500 (9)	
N2	0.51832 (12)	0.5539 (4)	0.41898 (14)	0.0359 (6)	
N3	0.57592 (12)	0.3670 (4)	0.41555 (14)	0.0348 (6)	
N4	0.66919 (15)	0.1462 (4)	0.35159 (18)	0.0478 (8)	
N5	0.65754 (15)	0.0517 (4)	0.42870 (17)	0.0464 (8)	
C1	0.30250 (19)	1.3389 (5)	0.3361 (2)	0.0539 (10)	
C2	0.37083 (16)	1.1579 (4)	0.32957 (17)	0.0396 (8)	
C3	0.40056 (18)	0.9771 (5)	0.37679 (18)	0.0415 (8)	
C4	0.46538 (15)	0.8755 (4)	0.33223 (17)	0.0350 (7)	
C5	0.52042 (14)	0.6671 (4)	0.35064 (17)	0.0342 (7)	
C6	0.61891 (16)	0.3354 (4)	0.34707 (18)	0.0381 (7)	
C7	0.60185 (15)	0.1833 (5)	0.46600 (18)	0.0388 (8)	
C8	0.61793 (19)	0.3882 (5)	0.6143 (2)	0.0488 (9)	
C9	0.70934 (17)	0.3711 (4)	0.60716 (16)	0.0391 (8)	
C10	0.7528 (2)	0.5388 (5)	0.5610 (2)	0.0523 (9)	
C11	0.8368 (2)	0.5174 (7)	0.5528 (3)	0.0639 (11)	
C12	0.8789 (2)	0.3276 (7)	0.5891 (2)	0.0603 (11)	
C13	0.8372 (2)	0.1623 (6)	0.6353 (3)	0.0628 (11)	
C14	0.7532 (2)	0.1828 (6)	0.6448 (2)	0.0556 (11)	
H1A	0.32400	1.49870	0.35070	0.0810*	
H1B	0.26440	1.28400	0.37630	0.0810*	
H1C	0.27480	1.35110	0.28550	0.0810*	
H3	0.38240	0.93020	0.42740	0.0500*	
H8A	0.59980	0.54590	0.59240	0.0590*	
H8B	0.60300	0.38470	0.67050	0.0590*	
H10	0.72510	0.66720	0.53530	0.0630*	
H11	0.86540	0.63330	0.52220	0.0770*	
H12	0.93530	0.31210	0.58220	0.0730*	
H13	0.86540	0.03440	0.66070	0.0750*	
H14	0.72560	0.06900	0.67690	0.0670*	

### Atomic displacement parameters (Å^2^)

**Table:**

	*U*^11^	*U*^22^	*U*^33^	*U*^12^	*U*^13^	*U*^23^
S1	0.0443 (3)	0.0412 (3)	0.0434 (4)	0.0021 (3)	0.0010 (3)	−0.0021 (3)
S2	0.0399 (3)	0.0460 (3)	0.0582 (4)	−0.0038 (3)	0.0005 (4)	0.0125 (3)
O1	0.0461 (11)	0.0513 (11)	0.0566 (15)	0.0077 (8)	−0.0012 (9)	0.0149 (10)
N1	0.0457 (13)	0.0496 (13)	0.0546 (18)	0.0096 (10)	0.0017 (11)	0.0107 (12)
N2	0.0286 (10)	0.0314 (10)	0.0476 (13)	0.0009 (7)	−0.0017 (9)	−0.0029 (9)
N3	0.0276 (9)	0.0299 (10)	0.0470 (13)	0.0001 (7)	−0.0024 (9)	−0.0055 (9)
N4	0.0436 (13)	0.0434 (12)	0.0564 (15)	0.0092 (10)	−0.0002 (12)	−0.0080 (11)
N5	0.0415 (12)	0.0367 (12)	0.0611 (16)	0.0062 (9)	−0.0050 (12)	−0.0029 (11)
C1	0.0434 (15)	0.0392 (14)	0.079 (2)	0.0064 (11)	−0.0022 (16)	0.0071 (15)
C2	0.0334 (12)	0.0312 (12)	0.0543 (17)	−0.0041 (9)	−0.0055 (11)	−0.0018 (12)
C3	0.0426 (14)	0.0345 (12)	0.0474 (17)	0.0023 (10)	−0.0002 (12)	0.0018 (12)
C4	0.0322 (11)	0.0280 (10)	0.0449 (15)	−0.0040 (8)	−0.0077 (10)	−0.0004 (10)
C5	0.0303 (11)	0.0272 (11)	0.0450 (15)	−0.0033 (8)	−0.0048 (11)	−0.0058 (10)
C6	0.0332 (12)	0.0334 (11)	0.0478 (15)	−0.0024 (9)	−0.0036 (12)	−0.0068 (11)
C7	0.0312 (12)	0.0342 (12)	0.0509 (16)	−0.0024 (9)	−0.0054 (11)	−0.0011 (11)
C8	0.0520 (17)	0.0423 (14)	0.0522 (18)	0.0040 (12)	0.0088 (14)	−0.0035 (13)
C9	0.0451 (14)	0.0368 (12)	0.0354 (14)	−0.0027 (10)	0.0008 (11)	−0.0051 (11)
C10	0.0605 (17)	0.0420 (14)	0.0544 (17)	−0.0026 (12)	−0.0032 (17)	0.0069 (14)
C11	0.059 (2)	0.0688 (19)	0.064 (2)	−0.0199 (16)	0.0027 (18)	0.0050 (18)
C12	0.0470 (17)	0.072 (2)	0.062 (2)	−0.0033 (15)	−0.0081 (15)	−0.0165 (18)
C13	0.057 (2)	0.0575 (19)	0.074 (2)	0.0046 (15)	−0.0232 (18)	0.0005 (17)
C14	0.0602 (19)	0.0467 (16)	0.060 (2)	−0.0040 (13)	−0.0082 (15)	0.0126 (15)

### Geometric parameters (Å, º)

**Table:**

S1—C5	1.753 (3)	C8—C9	1.495 (4)
S1—C6	1.739 (3)	C9—C10	1.382 (4)
S2—C7	1.738 (3)	C9—C14	1.390 (4)
S2—C8	1.835 (3)	C10—C11	1.378 (5)
O1—N1	1.401 (3)	C11—C12	1.371 (5)
O1—C2	1.348 (4)	C12—C13	1.359 (5)
N1—C4	1.305 (4)	C13—C14	1.380 (5)
N2—N3	1.376 (3)	C1—H1A	0.9600
N2—C5	1.294 (4)	C1—H1B	0.9600
N3—C6	1.351 (4)	C1—H1C	0.9600
N3—C7	1.366 (4)	C3—H3	0.9300
N4—N5	1.397 (4)	C8—H8A	0.9700
N4—C6	1.308 (3)	C8—H8B	0.9700
N5—C7	1.308 (4)	C10—H10	0.9300
C1—C2	1.482 (4)	C11—H11	0.9300
C2—C3	1.342 (4)	C12—H12	0.9300
C3—C4	1.402 (4)	C13—H13	0.9300
C4—C5	1.468 (3)	C14—H14	0.9300
			
C5—S1—C6	86.79 (13)	C10—C9—C14	117.8 (3)
C7—S2—C8	99.29 (14)	C9—C10—C11	120.6 (3)
N1—O1—C2	109.1 (2)	C10—C11—C12	120.9 (3)
O1—N1—C4	104.2 (2)	C11—C12—C13	119.3 (3)
N3—N2—C5	106.8 (2)	C12—C13—C14	120.5 (3)
N2—N3—C6	118.7 (2)	C9—C14—C13	121.0 (3)
N2—N3—C7	135.6 (2)	C2—C1—H1A	110.00
C6—N3—C7	105.7 (2)	C2—C1—H1B	109.00
N5—N4—C6	104.6 (2)	C2—C1—H1C	109.00
N4—N5—C7	109.6 (2)	H1A—C1—H1B	109.00
O1—C2—C1	115.7 (2)	H1A—C1—H1C	109.00
O1—C2—C3	109.6 (2)	H1B—C1—H1C	109.00
C1—C2—C3	134.7 (3)	C2—C3—H3	128.00
C2—C3—C4	104.0 (3)	C4—C3—H3	128.00
N1—C4—C3	113.0 (2)	S2—C8—H8A	109.00
N1—C4—C5	116.7 (2)	S2—C8—H8B	109.00
C3—C4—C5	130.2 (3)	C9—C8—H8A	109.00
S1—C5—N2	118.27 (17)	C9—C8—H8B	109.00
S1—C5—C4	119.8 (2)	H8A—C8—H8B	108.00
N2—C5—C4	121.9 (2)	C9—C10—H10	120.00
S1—C6—N3	109.44 (17)	C11—C10—H10	120.00
S1—C6—N4	138.7 (2)	C10—C11—H11	120.00
N3—C6—N4	111.9 (3)	C12—C11—H11	119.00
S2—C7—N3	124.7 (2)	C11—C12—H12	120.00
S2—C7—N5	127.1 (2)	C13—C12—H12	120.00
N3—C7—N5	108.2 (3)	C12—C13—H13	120.00
S2—C8—C9	112.91 (19)	C14—C13—H13	120.00
C8—C9—C10	120.9 (2)	C9—C14—H14	120.00
C8—C9—C14	121.3 (2)	C13—C14—H14	119.00
			
C5—S1—C6—N4	178.0 (3)	N5—N4—C6—N3	−0.3 (3)
C6—S1—C5—N2	−0.6 (2)	N5—N4—C6—S1	−178.5 (2)
C6—S1—C5—C4	−178.2 (2)	C6—N4—N5—C7	0.5 (3)
C5—S1—C6—N3	−0.21 (18)	N4—N5—C7—N3	−0.4 (3)
C8—S2—C7—N5	104.8 (3)	N4—N5—C7—S2	178.5 (2)
C8—S2—C7—N3	−76.5 (2)	C1—C2—C3—C4	178.8 (3)
C7—S2—C8—C9	−58.4 (2)	O1—C2—C3—C4	−0.1 (3)
N1—O1—C2—C1	−179.0 (2)	C2—C3—C4—C5	−178.1 (3)
C2—O1—N1—C4	−0.2 (3)	C2—C3—C4—N1	−0.1 (3)
N1—O1—C2—C3	0.1 (3)	C3—C4—C5—N2	−5.1 (4)
O1—N1—C4—C3	0.1 (3)	N1—C4—C5—S1	−5.5 (3)
O1—N1—C4—C5	178.5 (2)	N1—C4—C5—N2	176.9 (2)
C5—N2—N3—C6	−1.3 (3)	C3—C4—C5—S1	172.5 (2)
N3—N2—C5—C4	178.7 (2)	S2—C8—C9—C10	109.4 (3)
N3—N2—C5—S1	1.1 (3)	S2—C8—C9—C14	−68.8 (3)
C5—N2—N3—C7	−178.4 (3)	C8—C9—C10—C11	−178.1 (3)
N2—N3—C7—S2	−1.4 (4)	C14—C9—C10—C11	0.2 (5)
N2—N3—C6—N4	−177.8 (2)	C8—C9—C14—C13	177.4 (3)
C6—N3—C7—N5	0.2 (3)	C10—C9—C14—C13	−1.0 (5)
C7—N3—C6—N4	0.1 (3)	C9—C10—C11—C12	1.0 (6)
C7—N3—C6—S1	178.79 (17)	C10—C11—C12—C13	−1.6 (6)
N2—N3—C6—S1	0.9 (3)	C11—C12—C13—C14	0.9 (6)
N2—N3—C7—N5	177.6 (3)	C12—C13—C14—C9	0.4 (6)
C6—N3—C7—S2	−178.7 (2)		
