# Supplementary figures and images for: Crystal structure of 3-benzyl­sulfanyl-6-(5-methyl-1,2-oxazol-3-yl)-1,2,4-triazolo[3,4-b][1,3,4]thia­diazole
Source: Acta Crystallogr E Crystallogr Commun. 2015 Oct 3;71(Pt 11):o809–10. doi: 10.1107/S2056989015017351 (PMC4645033; doi:10.1107/S2056989015017351)

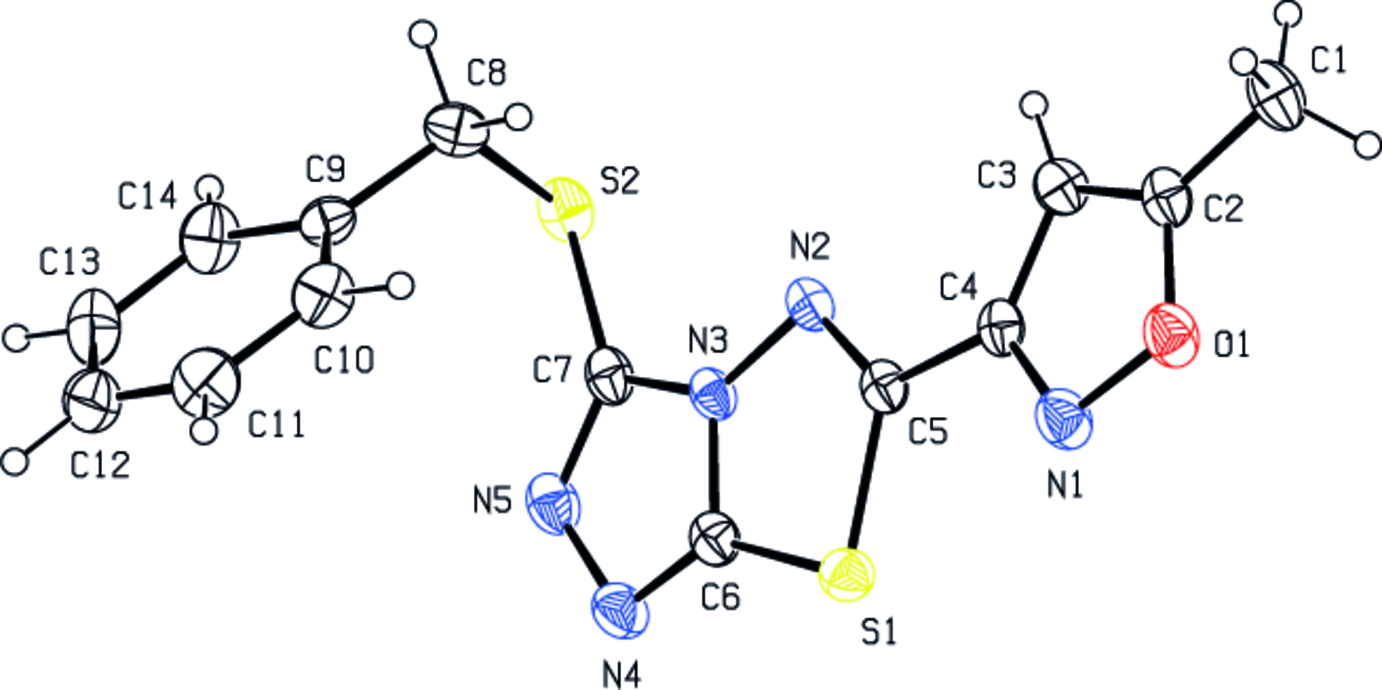

Supplement: Supplementary file 4 [file e-71-0o809-fig1.tif]

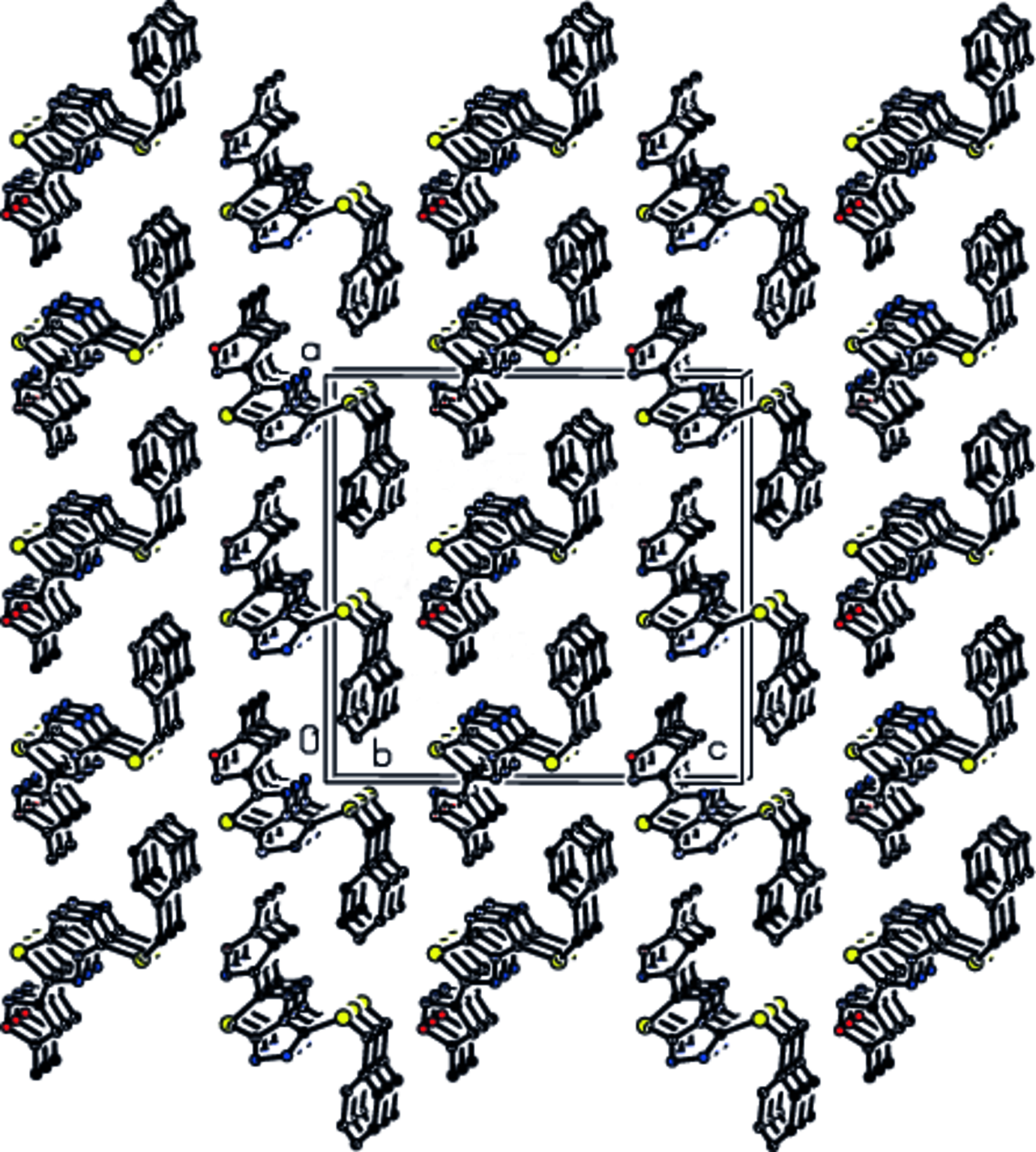

Supplement: Supplementary file 5 [file e-71-0o809-fig2.tif]
